# FRET-Based Aptasensor for the Selective and Sensitive Detection of Lysozyme

**DOI:** 10.3390/s20030914

**Published:** 2020-02-09

**Authors:** Kumar Sapkota, Soma Dhakal

**Affiliations:** Department of Chemistry, Virginia Commonwealth University, Richmond, VA 23284, USA; sapkotak@vcu.edu

**Keywords:** lysozyme, aptasensor, biomarker, fluorescence, single-molecule FRET, recyclable sensor

## Abstract

Lysozyme is a conserved antimicrobial enzyme and has been cited for its role in immune modulation. Increase in lysozyme concentration in body fluids is also regarded as an early warning of some diseases such as Alzheimer’s, sarcoidosis, Crohn’s disease, and breast cancer. Therefore, a method for a sensitive and selective detection of lysozyme can benefit many different areas of research. In this regard, several aptamers that are specific to lysozyme have been developed, but there is still a lack of a detection method that is sensitive, specific, and quantitative. In this work, we demonstrated a single-molecule fluorescence resonance energy transfer (smFRET)-based detection of lysozyme using an aptamer sensor (also called aptasensor) in which the binding of lysozyme triggers its conformational switch from a low-FRET to high-FRET state. Using this strategy, we demonstrated that the aptasensor is sensitive down to 2.3 picomoles (30 nM) of lysozyme with a dynamic range extending to ~2 µM and has little to no interference from similar biomolecules. The smFRET approach used here requires a dramatically small amount of aptasensor (~3000-fold less as compared to typical bulk fluorescence methods), and it is cost effective compared to enzymatic and antibody-based approaches. Additionally, the aptasensor can be readily regenerated in situ via a process called toehold mediated strand displacement (TMSD). The FRET-based aptasensing of lysozyme that we developed here could be implemented to detect other protein biomarkers by incorporating protein-specific aptamers without the need for changing fluorophore-labeled DNA strands.

## 1. Introduction

Lysozyme is an antimicrobial enzyme and is found in diverse organisms, including bacteria, fungi, plants, and mammals. Lysozyme has been used as a model protein in biotechnology and pharmaceutical industries to study enzyme catalysis and protein structure [1,2]. It is well known for its bacteriolytic activity, meaning that it destroys the cell wall of Gram-positive bacteria by catalyzing the cleavage of the β-1,4 glycosidic bond in peptidoglycan residues [3,4,5,6,7]. In addition, lysozyme possesses an anti-proliferative effect against cancer and lung fibroblasts [8,9]. Therefore, it is often referred as the “body’s own antibiotic” [1]. Although the typical concentration of lysozyme in serum is 2.8 ± 0.8 mg/L [10,11], its concentration in body fluids rises on the onset of several diseases such as AIDS [12], cancer [13], malaria [14], Alzheimer’s disease [15,16], meningitis [17], rheumatoid arthritis [18,19], sarcoidosis [11], and Crohn’s disease [20]. Thus, lysozyme serves as a biomarker for several diseases. Hence, monitoring its concentration can be useful for early-stage diagnosis of diseases. Therefore, sensitive and specific detection of lysozyme is helpful in biomarker analysis in clinics and in biotechnology.

A number of techniques have been demonstrated for the detection of lysozyme including classical analytical methods such as chromatography [21] and enzyme-linked immunosorbent assay (ELISA) [22,23]. Recent techniques for lysozyme analysis include electrochemical [2,24,25,26], optical [27,28,29], colorimetric [5,30,31], and surface plasmon resonance (SPR) [32,33], some of which are highly sensitive with detection limits in the picomolar to femtomolar ranges [1,2,24,26,27]. Nevertheless, most of these detection methods suffer from one or more problems such as low-selectivity, complex sample pre-treatment, time-consuming immobilizing processes, slow response time, etc. For example, the electrochemical detection typically requires time consuming electrode/surface preparation, complicated sensor immobilization processes, and/or labeling of the probe with a redox moiety such as ferrocene [25,26,34]. Other methods such as SPR usually require overnight or several days of surface fabrication and costly probes/reagents [32,33]. Optical and colorimetric assays typically require large amount of probe samples [29,30]. Although sensitive sensors are highly desirable, given the fact that the concentration of lysozyme in serum and plasma is in the nanomolar (nM) range or higher [10,11], the actual need is a simple and selective sensor with a large dynamic range that works with a small amount of sample. Motivated by this, here we developed a fluorescence resonance energy transfer (FRET)-based [35] single-step detection of lysozyme using an aptasensor, which has several advantages as noted below. 

Aptamers are short single-stranded sequences of nucleic acids (usually 10–100 nucleotides in length) that bind to their specific targets with high affinity and selectivity [36,37,38,39]. Therefore, aptamer-based detection of biomolecules is on the rise in recent years due to several benefits over antibody-based methods. For example, aptamer generation is significantly easier and cheaper than antibody production [36,37,38,39]. Aptamers are virtually non-immunogenic and also have a longer shelf-life and higher thermal stability than antibodies. In addition, aptamers have better access to the target molecules owing to their flexibility and small size [40,41]. Thus, they are suitable recognition elements for the detection of protein biomarkers such as lysozyme [24,26,34]. Herein, using a lysozyme-specific aptamer in a single-molecule FRET (smFRET) platform [42,43,44]. we demonstrated a single-step detection of lysozyme on a recyclable platform. 

The lysozyme sensing strategy developed here has several advantages. For example, the smFRET approach needs ~3000-fold less sample amount than bulk fluorescence approaches (~100 µL of ~20 pM aptasensor in single molecule vs. ~200 µL of ~30–60 nM probes in traditional bulk fluorescence). The aptasensor is recyclable within a few minutes by an in situ toehold mediated strand displacement (TMSD) process [45]. All of the DNA strands including the fluorophore-labeled strands are readily available by custom-synthesis from many companies, there is no need for complex sample pre-treatment steps, and simply mixing constituent DNAs and thermal annealing is sufficient to run experiments for 2–3 weeks. Therefore, the sensing strategy developed here has the potential to be highly useful for detection of lysozyme in clinics, the food industry, and in many other biotechnological applications. 

## 2. Materials and Methods

### 2.1. Chemicals

Most of the chemicals—including magnesium chloride hexahydrate, protocatechuate 3,4-dioxygenase (PCD), 6-hydroxy-2,5,7,8-tetramethylchroman-2-carboxylic acid (Trolox), tris(hydroxymethyl)-aminomethane (Tris), ethylenediaminetetraacetic acid disodium salt (EDTA), and acetic acid—were purchased from Fisher Scientific. Biotinylated bovine serum albumin (bBSA) was purchased from Thermo Scientific, dispersed in filtered sterile water to a concentration of 1 mg/mL, and stored at −20 °C until used. Protocatechuic acid (PCA), streptavidin, lysozyme from chicken egg white (MW 14.3 kDa), bovine serum albumin (BSA), and D-glucose anhydrous were purchased from VWR. Cytochrome C (Cyt-C) from bovine heart was obtained from Sigma Aldrich. All DNA oligonucleotides (nts), including a strand with 30-nucleotides lysozyme aptamer [46], were purchased from Integrated DNA Technologies (IDT Inc.) and stored at −20 °C until needed. 

### 2.2. Preparation of Aptasensor

Lysozyme aptasensor was assembled by thermal annealing of the constituent single-stranded DNA (ssDNA) oligonucleotides (nts) along with a lysozyme aptamer (5′-ACT GTC **ATC AGG GCT AAA GAG TGC AGA GTT ACT TAG**, Supplementary Materials Table S1) pre-mixed at 1 µM concentrations in 1× TAE-Mg buffer (40 mM Tris, 20 mM acetic acid, 1 mM EDTA, 10 mM Mg^2+^, pH 7.4). The lysozyme aptamer is shown in bold with a 6-nucleotide extension at the 5′-end. Thermal annealing was carried out by ramping the temperature of the solution from 95 to 4 °C in a thermal cycler as described in our previous publications [47]. Then the annealed sample was stored at 4 °C until needed. The full assembly of the aptasensor was verified by running a native 7.5% polyacrylamide gel electrophoresis (PAGE) (Supplementary Materials Figure S1).

### 2.3. Preparation of Functionalized Flow Cell

Flow cells were prepared using standard microscope quartz slides and cover slips as described in our previous work [48]. In order to enable surface-tethering of aptasensor molecules, each flow cell was incubated with 1 mg/mL biotinylated BSA (bBSA) for 5 min followed by 0.2 mg/mL streptavidin for 2 min. In this process, bBSA first binds to the quartz slide via non-specific adsorption. The adsorption of bBSA on the microscope slide provides a surface for streptavidin binding via affinity interaction between biotin and streptavidin and also helps passivate the surface to reduce any non-specific binding of DNA and protein to the slide. Then the excess streptavidin was flushed with 1× TAE-Mg buffer.

### 2.4. Aptasensor Immobilization and Single-Molecule Imaging

The functionalized flow cell was mounted on the stage of a custom-built prism-based total internal reflection fluorescence (pTIRF) microscope [44]. Then, a 20 pM aptasensor solution that was prepared in 1× TAE imaging buffer consisting of 10 mM MgCl_2_ and an oxygen scavenging system (4 mM Trolox, 10 mM PCA, 100 nM PCD) was injected into the flow cell and incubated for ~30 s. The flow cell was then flushed to remove the unbound molecules using the imaging buffer. Subsequently, 1 μM H1 strand along with lysozyme at different concentrations were prepared in the imaging buffer, injected, and incubated in the flow cell for 20 min before recording the movies. Movies were recorded using Single.exe software as described [44,49]. The Cy3 fluorophore was continuously excited with a 532 nm He–Ne laser, and the resulting fluorescence emissions of both the Cy3 and Cy5 fluorophores were concurrently recorded through green and red channels (512 × 256 pixels) using an EMCCD camera (iXon 897, Andor) with 100 ms time resolution. The presence of an active FRET pair was confirmed toward the end of each movie by turning on a 639 nm red laser. All single-molecule experiments were performed at room temperature (23 °C).

### 2.5. Single-Molecule Data Analysis

Acquired movies from the single molecule fluorescence experiments were processed, and fluorescence–time trajectories of individual aptasensor molecules were obtained using IDL and MATLAB scripts available from the Ha Lab [50]. Single molecules exhibiting a clear evidence for the presence of both Cy3 and Cy5 fluorophores and a single-step photo-bleaching of the fluorophores were manually picked using MATLAB program. The first 60 frames of data of the selected single-molecule FRET traces were combined in Origin, and the FRET efficiency (*E*_FRET_) value was calculated using the equation *I*_A_/(*I*_D_ + *I*_A_), where *I*_A_ and *I*_D_ represent the background-corrected fluorescence intensities of the acceptor and donor fluorophores, respectively [42,51]. FRET efficiency histograms were made, and *E*_FRET_ and area under the curve (AUC) were determined by fitting the FRET histograms with a single or multi-peak Gaussian function. Standard deviation (σ) in the FRET efficiency and AUC were determined from three FRET histograms after randomly assigning the molecules from each experimental condition into three groups.

## 3. Results and Discussion

### 3.1. Experimental Design

**Figure 1:**
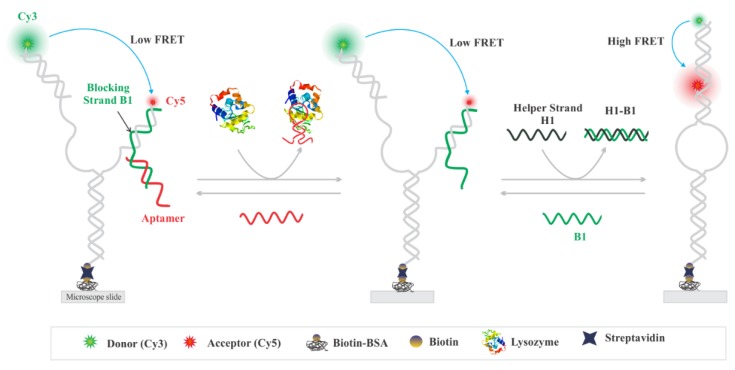
Sensor design and working principle. Sensor design with the fluorophore labeling scheme (Cy3: green and Cy5: red). The microscope slide was modified with biotinylated bovine serum albumin (BSA) and then with streptavidin to allow surface-immobilization of the biotinylated aptasensor via the biotin–streptavidin interaction. Lysozyme-specific aptamer prevents the helper strand (H1) from binding to the blocker strand (B1). When lysozyme takes the aptamer away from the sensor molecule, the toehold region of the B1 strand is exposed. This allows toehold-mediated displacement of B1, forming a B1–H1 duplex as a byproduct. This process allows the aptasensor molecules to adopt a closed conformation, leading to a significantly higher fluorescence resonance energy transfer (FRET) efficiency than that of the open conformation.

The design and working principle of the lysozyme aptasensor is illustrated in Figure 1. The aptasensor was prepared by thermal annealing of single-stranded DNA (ssDNA) oligonucleotides (Supplementary Materials Table S1 and Figure 1) in 1 × TAE-Mg buffer (40 mM Tris, 20 mM acetic acid, 1 mM EDTA, 10 mM Mg^2+^, pH 7.4) by ramping the temperature from 95 to 4 °C as described previously [46,48]. The aptasensor is composed of two partially complementary DNA arms, each labeled with either a donor or an acceptor fluorophore to enable FRET when the complementary arms hybridize to one another. One of the arms carries a blocker strand (B1), which is extended by 15 nts to partially hybridize with a lysozyme aptamer [46]. In the absence of lysozyme, the aptasensor remains in an open state yielding little to no FRET efficiency. Here the lysozyme aptamer was incorporated into the aptasensor in a way that a significant portion (9 nts) of the aptamer was blocked by binding to a blocker (B1) strand (Figure 1). For this, the aptamer sequence was extended at its 5′-end by 6 nts (ACT GCT) so that the extended 6 nts along with 9 nts aptamer (total of 15 nts) stably hybridized with the B1 strand. This design serves two purposes: First, it allows incorporation of lysozyme aptamer to the sensor. Second, it blocks the helper strand (H1) from binding to strand B1 in the absence of lysozyme. In this design, when a mixture of H1 strand and lysozyme are added to the aptasensor, lysozyme binds to the aptamer and the aptamer is displaced from the sensor allowing toehold-mediated displacement of B1 by H1. For an efficient removal of the blocker strand, an excess of H1 strand (1 µM) was used in the imaging solution. This approach allows lysozyme-dependent conformational switching of the aptasensor enabling a higher FRET efficiency. 

In order to allow FRET measurement, the distal ends of oligonucleotide arms are labeled with either a donor (Cy3) or an acceptor (Cy5) fluorophore, so that the extent of energy transfer can be directly probed by measuring the FRET efficiency (*E*_FRET_). When the aptasensor is in the open conformation, the FRET efficiency is little to none due to spatial separation of the FRET pair (Figure 1). In our design, the complementary sequences are blocked by duplex formation to avoid hybridization between Cy3- and Cy5-labeled arms in the absence of target. However, in the presence of lysozyme, the aptamer dissociates from the sensor due to the formation of a lysozyme–aptamer complex. The removal of the aptamer from the sensor molecule allows unzipping of the B1 strand by H1 via TMSD, which ultimately allows the labeled strands to hybridize resulting in a high-FRET.

### 3.2. Single-Molecule Analysis of Aptasensor

Aptasensor molecules were immobilized on the microscope slide using the biotin/streptavidin interaction (Figure 1) as described above in the Materials and Methods [46,48]. Briefly, a 20 pM aptasensor solution was injected into the flow cell and incubated for less than a minute. The unbound molecules were washed away by flushing an imaging buffer containing an oxygen scavenging system (OSS), which served to curtail fluorophore blinking and photobleaching upon laser illumination [49,52]. The flow cell was then irradiated with a 532 nm laser to create an evanescent field, which excites the Cy3 fluorophores of surface-tethered molecules [49]. The excited Cy3 fluorophores transfer energy to the Cy5 fluorophore present in the same molecule via dipole–dipole coupling, a process known as fluorescence resonance energy transfer (FRET) [35]. The fluorescence emissions of both the fluorophores, Cy3 and Cy5, were recorded at 10 frames per second (~100 ms camera integration time, gain 200). The presence of Cy5 fluorophore was confirmed by direct excitation using a red laser toward the end of the movies. The fluorescence movies were processed with IDL and MATLAB codes (see Materials and Methods). Only those molecular traces that showed the presence of both fluorophores were selected for further data analysis, and the FRET histograms were plotted using the Origin software. The total time per analysis from single molecule experiments to data analysis was approximately 2–3 hr.

**Figure 2:**
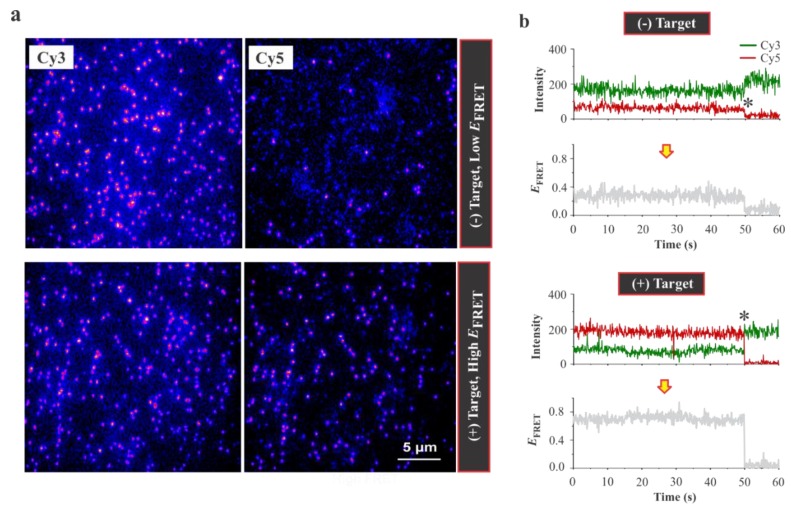
Single molecule visualization of lysozyme binding. (**a**) Fluorescence snap-shots of the surface-immobilized aptasensors before (top) and after adding 0.3 μM lysozyme (bottom). The Cy5 channel lit up significantly after adding lysozyme, which demonstrated an increase in the FRET efficiency due to lysozyme dependent conformational switch of the aptasensors. Scale bar 5 µm. (**b**) Typical single molecule traces. Top: Open conformation (low-FRET efficiency (*E*_FRET_) state); Bottom: Closed conformation (high-*E*_FRET_ state). The fluorescence intensities of the donor (Cy3: green) and the accepter (Cy5: red) fluorophore and the corresponding FRET efficiencies are shown for both conformations. The asterisks indicate the photobleaching event of the Cy5 fluorophores. All of the experiments were performed in 1×TAE-Mg buffer at 23 °C.

Figure 2 shows the fluorescence field of views of Cy3 and Cy5 channels, typical fluorescence–time trajectories, and corresponding FRET traces before and after adding lysozyme. Significant increase in the number of molecules with Cy5 emission after addition of lysozyme demonstrated that the high FRET is lysozyme-dependent (Figure 2a). As expected, the single molecule traces acquired from these movies showed a high and low intensity for Cy3 and Cy5 emissions, respectively, in the absence of target. However, the intensities were switched (Cy5 was higher than Cy3) after adding the target (Figure 2b), demonstrating a lysozyme-dependent FRET.

Using this strategy, we first demonstrated that the aptasensor can be recycled (Supplementary Materials Figure S2). We also demonstrated that the FRET results are reproducible and are suitable for detecting a biologically relevant concentration of lysozyme [4,10]. The aptasensor can be used for up to three weeks by storing at 4 °C or annealed fresh, as only a little sample is needed for single-molecule experiments. The stem between the internal bulge and the Cy5-labeled nucleotide was optimized to be 6 bp to allow full recycling of aptasensors for multiple rounds of detection [49].

### 3.3. Design Feasibility Assessment

**Figure 3:**
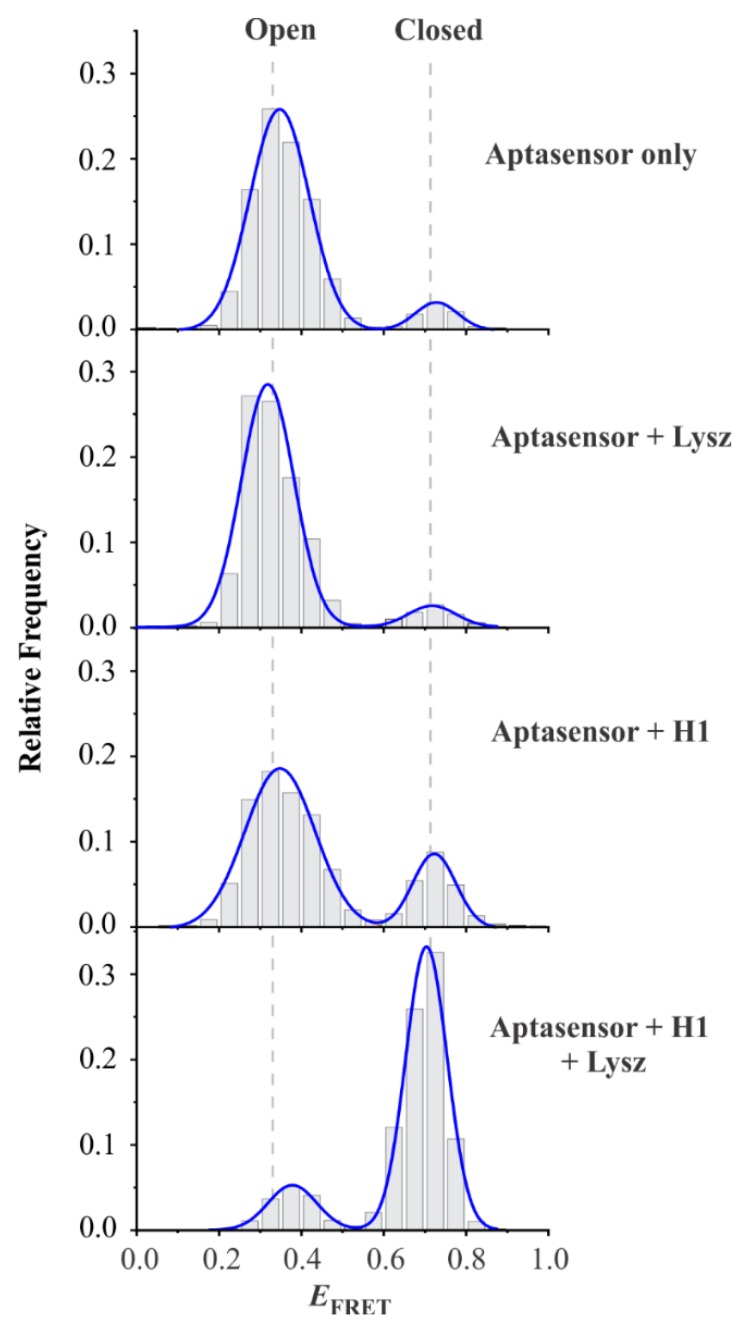
Lysozyme sensing. FRET efficiency histograms of “Aptasensor”, “Aptasensor + lysozyme”, “aptasensor + H1”, and “aptasensor + H1 + Lysozyme”, respectively (from top to bottom). The term “Lysz” represents lysozyme. The concentration of aptasensor, H1, and lysozyme used were 20 pM, 1 µM, and 2 µM, respectively. The data show that both lysozyme and H1 are required for high-FRET signal. Each histogram is prepared from more than 100 molecules.

To determine the sensing ability of the lysozyme aptasensor, a series of *E*_FRET_ histograms were acquired under different conditions as demonstrated in Figure 3. As expected, the aptasensor alone (in the absence of lysozyme and helper strand H1) showed primarily a low-*E*_FRET_ population with a FRET value of ~0.35. Adding lysozyme (Aptasensor + Lysz) did not show any increase in the high-*E*_FRET_ population, demonstrating that the lysozyme alone was unable to change the aptasensor conformation. This result was expected because the H1 strand has to pull off the B1 strand first to switch the sensor from low- to high-*E*_FRET_, which was not possible in the absence of H1. In both cases (Aptasensor alone and Aptasensor + Lysz), we observed a small fraction (~8%) of high-FRET state, which we attributed to the sensor molecules missing the blocker strand B1. Similarly, in the presence of helper H1 (Aptasensor + H1) without lysozyme, we observed further increase (by ~15%) in the high-*E*_FRET_ fraction. This observation suggested that the aptamer sequence is missing in a small fraction of aptasensor, leading to TMSD of B1 by H1. 

However, the presence of a small fraction of high-*E*_FRET_ state does not interfere with lysozyme detection and quantification, as one can solely rely on the increase in the high-*E*_FRET_ fraction above this background for an actual signal. In fact, in the presence of all components including lysozyme, a significant high-*E*_FRET_ fraction (84.3%) was observed. These observations demonstrated that our aptasensor is capable of detecting lysozyme.

### 3.4. Analytical Sensitivity

**Figure 4:**
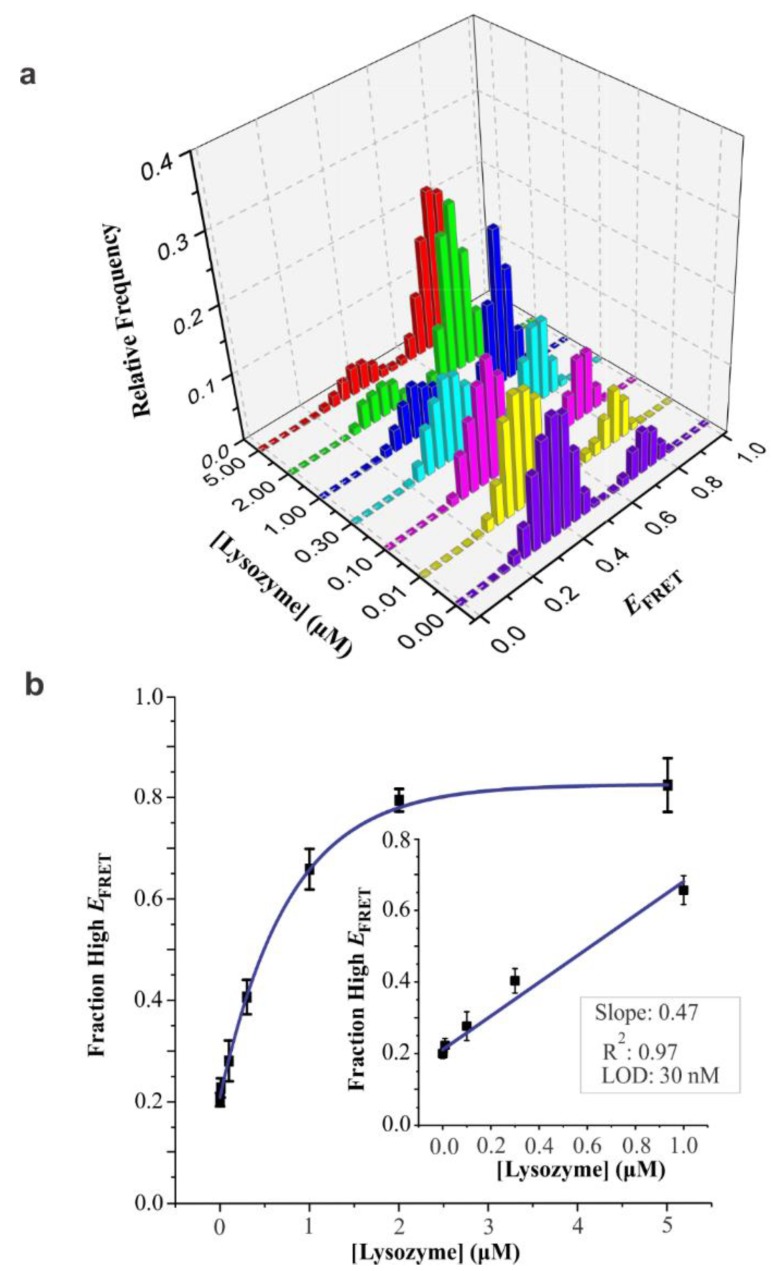
Analytical sensitivity of lysozyme aptasensor. (**a**) Single-molecule FRET (smFRET) histograms at various concentrations of lysozyme (0, 0.01, 0.10, 0.30, 1.00, 2.00, and 5.00 µM). (**b**) Standard curve obtained by plotting the fraction of high-*E*_FRET_ population versus lysozyme concentration. High-*E*_FRET_ population was determined from the two-peak Gaussian fitting of the histograms in Figure 4a. Inset depicts the linear region of the full curve, yielding R^2^ value of 0.97 and limit of detection (LOD) of 30 nM. Error bars represent the standard deviations (σ), n = 3. *E*_FRET_ histograms were prepared from >100 molecules at each concentration of lysozyme.

The analytical sensitivity of the lysozyme aptasensor was determined similarly as described in the Methods section above. Briefly, we acquired a series of smFRET histograms at different concentrations of lysozyme (0 to 5.0 µM) (Figure 4a). When we compared the area under the curve (AUC) of the high-*E*_FRET_ population to that of the low-*E*_FRET_ population at various concentrations of lysozyme, we observed a positive correlation between the high-*E*_FRET_ fraction and the concentration of target up to around 2 µM after which the curve plateaued (Figure 4b). The calculated limit of detection (LOD) is 30 nM (3σ) (Figure 4b, inset), which is 4.5 to 8-fold lower than the reported concentration of lysozyme in serum (2.8 ± 0.8 mg/L ~ 190.5 ± 54 nM) [10,53]. Given the flow cell volume of ~75 µL, the detection limit of 30 nM translates into 2.3 picomoles. This detection amount is comparable to other fluorescence-based methods for lysozyme detection (Supplementary Materials Table S3). In addition, our approach has a large dynamic range extending to 2 µM.

### 3.5. Selectivity of Lysozyme Aptasensor

Selectivity towards an intended target is one of the critical requirements for a sensor. Therefore, we went on to determine the selectivity of the lysozyme sensor by characterizing its performance in the presence of various biomolecules individually and in a mixture. For this purpose, we selected previously reported interfering biomolecules: bovine serum albumin (BSA), glucose, and cytochrome C (Cyt-C) separately and in a mixture [24,25,26,30]. To make this study relevant to the biological context, concentrations of each of the interfering biomolecules were kept similar to their biological concentrations [54,55,56]. 

**Figure 5:**
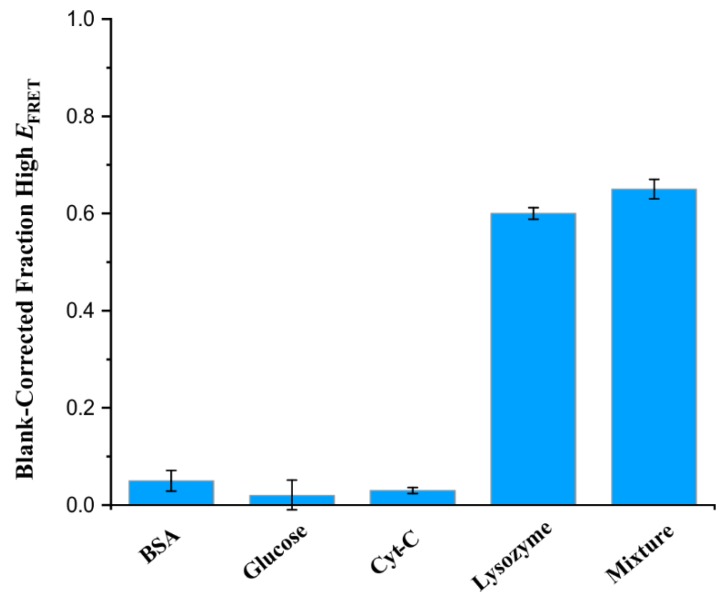
Selectivity test of the lysozyme aptasensor. The fraction of high-*E*_FRET_ in the presence of potential binder/interfering agents at their biologically relevant concentrations. The specificity was tested against BSA (1 mM), glucose (5.5 mM), cytochrome C (Cyt-C, 150 nM), Lysozyme (2 μM), and in a mixture of all targets (“Mixture”) at those concentrations. The fraction of the high-*E*_FRET_ population for each of the conditions was determined from two-peak Gaussian fitting of the smFRET histograms and corrected for the blank. Error bars indicate standard deviation determined from three groups of molecules.

In this study, while the high-*E*_FRET_ population for lysozyme was ~80% (after blank correction ~60%), the population in the presence of BSA, glucose, or cytochrome C was similar to the background, demonstrating a high selectivity of the aptasensor towards lysozyme (Figure 5). Additionally, the selectivity was also demonstrated when the aptasensor was tested in a mixture containing all of the interfering biomolecules.

## 4. Conclusions

Lysozyme serves as a biomarker for many diseases, so its detection and quantification are very important in clinical diagnostics. Despite several methods already available for lysozyme detection, a vast majority of them require complicated experimental design, expensive enzymes, or labeling of lysozyme in order to achieve a sensitive and specific detection. We developed an aptamer-based recyclable aptasensor, which allows the detection of lysozyme down to 2.3 picomoles with a large dynamic range extending to 2 µM. Further, the sensitivity and selectivity of the lysozyme aptasensor were verified in the presence of potential interfering agents. Therefore, recyclable aptasensors with a straightforward design and that provide a sensitive and selective one-step detection of lysozyme may find applications in quantitative analysis of lysozyme in various settings. Further, the developed strategy is generic and can be implemented to detect other protein biomarkers by incorporating protein-specific aptamers without the need for changing fluorophore-labeled DNA strands.

## Supplementary Materials

The following are available online at https://www.mdpi.com/1424-8220/20/3/914/s1: Table S1: DNA sequences, Table S2: Thermal annealing program for the assembly of lysozyme-specific aptasensor, Table S3: comparison of the analytical performance of lysozyme sensors, Figure S1: Native PAGE gel characterization, and Figure S2: Recyclability of the aptasensor.

## References

[1] Vasilescu A., Wang Q., Li M., Boukherroub R., Szunerits S. (2016). Aptamer-Based Electrochemical Sensing of Lysozyme. Chemosensors.

[2] Jamei H.R., Rezaei B., Ensafi A.A. (2019). An ultrasensitive electrochemical anti-lysozyme aptasensor with biorecognition surface based on aptamer/amino-rGO/ionic liquid/amino-mesosilica nanoparticles. Colloids Surf. B Biointerfaces.

[3] Ragland S.A., Criss A.K. (2017). From bacterial killing to immune modulation: Recent insights into the functions of lysozyme. PLoS Pathog..

[4] Khan N.I., Maddaus A.G., Song E. (2018). A Low-Cost Inkjet-Printed Aptamer-Based Electrochemical Biosensor for the Selective Detection of Lysozyme. Biosensors.

[5] Chen L., Xia N., Li T., Bai Y., Chen X. (2016). Aptasensor for visual and fluorometric determination of lysozyme based on the inner filter effect of gold nanoparticles on CdTe quantum dots. Microchim. Acta.

[6] Luo R., Zhou X., Chen Y., Tuo S., Jiang F., Niu X., Pan F., Wang H. (2019). Lysozyme Aptamer-Functionalized Magnetic Nanoparticles for the Purification of Lysozyme from Chicken Egg White. Foods.

[7] Pushkaran A.C., Nataraj N., Nair N., Götz F., Biswas R., Mohan C.G. (2015). Understanding the Structure–Function Relationship of Lysozyme Resistance in *Staphylococcus aureusby* Peptidoglycan O-Acetylation Using Molecular Docking, Dynamics, and Lysis Assay. J. Chem. Inf. Model..

[8] Khan I., Dowarha D., Katte R., Chou R.-H., Filipek A., Yu C. (2019). Lysozyme as the anti-proliferative agent to block the interaction between S100A6 and the RAGE V domain. PLoS ONE.

[9] Mahanta S., Paul S., Srivastava A., Pastor A., Kundu B., Chaudhuri T.K. (2015). Stable self-assembled nanostructured hen egg white lysozyme exhibits strong anti-proliferative activity against breast cancer cells. Colloids Surf. B Biointerfaces.

[10] Johansson B.G., Malmquist J. (1971). Quantitative Immunochemical Determination of Lysozyme (Muramidase) in Serum and Urine. Scand. J. Clin. Lab. Investig..

[11] Tomita H., Sato S., Matsuda R., Sugiura Y., Kawaguchi H., Niimi T., Yoshida S., Morishita M. (1999). Serum lysozyme levels and clinical features of sarcoidosis. Lung.

[12] Grieco M.H., Reddy M.M., Kothari H.B., Lange M., Buimovici-Klein E., William D. (1984). Elevated β2-microglobulin and lysozyme levels in patients with acquired immune deficiency syndrome. Clin. Immunol. Immunopathol..

[13] Serra C., Vizoso F., Alonso L., Rodríguez J.C., González L.O., Fernández M., Lamelas M.L., Sánchez L.M., García-Muñiz J.L., Baltasar A. (2002). Expression and prognostic significance of lysozyme in male breast cancer. Breast Cancer Res..

[14] Polimeni M., Valente E., Aldieri E., Khadjavi A., Giribaldi G., Prato M. (2012). Human lysozyme as a potential diagnostic marker in malaria: A mechanistic study of haemozoin-induced monocyte degranulation. Malar. J..

[15] Helmfors L., Boman A., Civitelli L., Nath S., Sandin L., Janefjord C., McCann H., Zetterberg H., Blennow K., Halliday G. (2015). Protective properties of lysozyme on β-amyloid pathology: Implications for Alzheimer disease. Neurobiol. Dis..

[16] Sandin L., Nath S., Armstrong A., Janefjord C., McCann H., Halliday G.M., Blennow K., Zetterberg H., Brorsson A.-C., Kagedal K. (2015). The Role of Lysozyme in Alzheimer’s Disease. Alzheimers Dement.

[17] Mishra O.P., Batra P., Ali Z., Anupurba S., Das B.K. (2003). Cerebrospinal fluid lysozyme level for the diagnosis of tuberculous meningitis in children. J. Trop. Pediatr..

[18] Pruzanski W., Saito S., Ogryzlo M.A. (1970). The significance of lysozyme (muramidase) in rheumatoid arthritis. i. levels in serum and synovial fluid. Arthritis Rheum..

[19] Torsteinsdóttir I., Håkansson L., Hällgren R., Gudbjörnsson B., Arvidson N.-G., Venge P. (1999). Serum lysozyme: A potential marker of monocyte/macrophage activity in rheumatoid arthritis. Rheumatology.

[20] Falchuk K.R., Perrotto J.L., Isselbacher K.J. (1975). Serum Lysozyme in Crohn’s Disease. A Useful Index of Disease Activity. Gastroenterology.

[21] Daeschel M.A., Musafija-Jeknic T., Wu Y., Bizzarri D., Villa A. (2002). High-Performance Liquid Chromatography Analysis of Lysozyme in Wine. Am. J. Enol. Vitic..

[22] Carstens C., Deckwart M., Webber-Witt M., Schäfer V., Eichhorn L., Brockow K., Fischer M., Christmann M., Paschke-Kratzin A. (2014). Evaluation of the Efficiency of Enological Procedures on Lysozyme Depletion in Wine by an Indirect ELISA Method. J. Agric. Food Chem..

[23] Kerkaert B., Mestdagh F., De Meulenaer B. (2010). Detection of hen’s egg white lysozyme in food: Comparison between a sensitive HPLC and a commercial ELISA method. Food Chem..

[24] Chen Z., Xu Q., Tang G., Liu S., Xu S., Zhang X. (2019). A facile electrochemical aptasensor for lysozyme detection based on target-induced turn-off of photosensitization. Biosens. Bioelectron..

[25] Ortiz-Aguayo D., Del Valle M. (2018). Label-Free Aptasensor for Lysozyme Detection Using Electrochemical Impedance Spectroscopy. Sensors.

[26] Xia Y., Gan S., Xu Q., Qiu X., Gao P., Huang S. (2013). A three-way junction aptasensor for lysozyme detection. Biosens. Bioelectron..

[27] Liu H., Zhang Y., Dong Y., Chu X. (2019). Electrogenerated chemiluminescence aptasensor for lysozyme based on copolymer nanospheres encapsulated black phosphorus quantum dots. Talanta.

[28] Fang M., Zhuo K., Chen Y., Zhao Y., Bai G., Wang J. (2019). Fluorescent probe based on carbon dots/silica/molecularly imprinted polymer for lysozyme detection and cell imaging. Anal. Bioanal. Chem..

[29] Zuo L., Qin G., Lan Y., Wei Y., Dong C. (2019). A turn-on phosphorescence aptasensor for ultrasensitive detection of lysozyme in humoral samples. Sens. Actuators B Chem..

[30] Lou T., Qiang H., Chen Z. (2017). Core-shell Cu@Au nanoparticles-based colorimetric aptasensor for the determination of lysozyme. Talanta.

[31] Chen Y.-M., Yu C.-J., Cheng T.-L., Tseng W.-L. (2008). Colorimetric Detection of Lysozyme Based on Electrostatic Interaction with Human Serum Albumin-Modified Gold Nanoparticles. Langmuir.

[32] Vasilescu A., Gáspár S., Gheorghiu M., David S., Dinca V., Peteu S., Wang Q., Li M., Boukherroub R., Szunerits S. (2017). Surface Plasmon Resonance based sensing of lysozyme in serum on Micrococcus lysodeikticus-modified graphene oxide surfaces. Biosens. Bioelectron..

[33] Mihai I., Vezeanu A., Polonschii C., Albu C., Radu G.-L., Vasilescu A. (2015). Label-free detection of lysozyme in wines using an aptamer based biosensor and SPR detection. Sens. Actuators B Chem..

[34] Cheng A.K.H., Ge B., Yu H.-Z. (2007). Aptamer-Based Biosensors for Label-Free Voltammetric Detection of Lysozyme. Anal. Chem..

[35] Sekar R.B., Periasamy A. (2003). Fluorescence resonance energy transfer (FRET) microscopy imaging of live cell protein localizations. J. Cell Boil..

[36] Nimse S.B., Sonawane M.D., Song K.-S., Kim T. (2016). Biomarker detection technologies and future directions. Analyst.

[37] Kaur H., Bruno J.G., Kumar A., Sharma T.K. (2018). Aptamers in the Therapeutics and Diagnostics Pipelines. Theranostics.

[38] Chikkaveeraiah B.V., Bhirde A.A., Morgan N.Y., Eden H.S., Chen X. (2012). Electrochemical Immunosensors for Detection of Cancer Protein Biomarkers. ACS Nano.

[39] Sun H., Zu Y. (2015). A Highlight of Recent Advances in Aptamer Technology and Its Application. Molecules.

[40] Wang G., Liu J., Chen K., Xu Y., Liu B., Liao J., Zhu L., Hu X., Li J., Pu Y. (2017). Selection and characterization of DNA aptamer against glucagon receptor by cell-SELEX. Sci. Rep..

[41] Pei X., Zhang J., Liu J. (2014). Clinical applications of nucleic acid aptamers in cancer. Mol. Clin. Oncol..

[42] Ha T. (2001). Single-Molecule Fluorescence Resonance Energy Transfer. Methods.

[43] Yang K., Yang Y., Zhang C.-Y. (2014). Single-molecule FRET for Ultrasensitive Detection of Biomolecules. NanoBioImaging.

[44] Gibbs D.R., Kaur A., Megalathan A., Sapkota K., Dhakal S. (2018). Build Your Own Microscope: Step-By-Step Guide for Building a Prism-Based TIRF Microscope. Methods Protoc..

[45] Srinivas N., Ouldridge T.E., Šulc P., Schaeffer J.M., Yurke B., Louis A.A., Doye J.P.K., Winfree E. (2013). On the biophysics and kinetics of toehold-mediated DNA strand displacement. Nucleic Acids Res..

[46] Cox J., Ellington A.D. (2001). Automated selection of anti-protein aptamers. Bioorganic Med. Chem..

[47] Kaur A., Sapkota K., Dhakal S. (2019). Multiplexed Nucleic Acid Sensing with Single-Molecule FRET. ACS Sens..

[48] Megalathan A., Cox B.D., Wilkerson P.D., Kaur A., Sapkota K., Reiner J.E., Dhakal S. (2019). Single-molecule analysis of i-motif within self-assembled DNA duplexes and nanocircles. Nucleic Acids Res..

[49] Sapkota K., Megalathan A., Moore D., Dhakal S., Kaur A., Donkoh-Moore C. (2019). Single-Step FRET-Based Detection of Femtomoles DNA. Sensors.

[50] Center for the Physics of Living Cells smFRET Data Acquisition and Analysis Package. https://cplc.illinois.edu/software/.

[51] Roy R., Hohng S., Ha T. (2008). A Practical Guide to Single Molecule FRET. Nat. Methods.

[52] Aitken C.E., Marshall R.A., Puglisi J.D. (2008). An Oxygen Scavenging System for Improvement of Dye Stability in Single-Molecule Fluorescence Experiments. Biophys. J..

[53] Porstmann B., Jung K., Schmechta H., Evers U., Pergande M., Porstmann T., Kramm H.-J., Krause H. (1989). Measurement of lysozyme in human body fluids: Comparison of various enzyme immunoassay techniques and their diagnostic application. Clin. Biochem..

[54] Rubio-Ruiz M.E., Díaz-Díaz E., Cárdenas-León M., Argüelles-Medina R., Sánchez-Canales P., Larrea-Gallo F., Soria-Castro E., Guarner-Lans V. (2008). Glycation does not modify bovine serum albumin (BSA)-induced reduction of rat aortic relaxation: The response to glycated and nonglycated BSA is lost in metabolic syndrome. Glycobiology.

[55] Fukumori Y., Takeda H., Fujisawa T., Ushijima K., Onodera S., Shiomi N. (2000). Blood glucose and insulin concentrations are reduced in humans administered sucrose with inosine or adenosine. J. Nutr..

[56] Eleftheriadis T., Pissas G., Liakopoulos V., Stefanidis I. (2016). Cytochrome c as a Potentially Clinical Useful Marker of Mitochondrial and Cellular Damage. Front. Immunol..

