# Supplementary material for: FRET-Based Aptasensor for the Selective and Sensitive Detection of Lysozyme
Source: Sensors (Basel). 2020 Feb 9;20(3):914. doi: 10.3390/s20030914 (PMC7038949; doi:10.3390/s20030914)
Supplement: Supplementary file 1 [file sensors-20-00914-s001.pdf]

sensors

Supporting Information

FRET-Based Aptasensor for the Selective and Sensitive Detection of Lysozyme

Kumar Sapkota and Soma Dhakal *

Department of Chemistry, Virginia Commonwealth University, Richmond, VA 23284, USA; sapkotak@vcu.edu

* Correspondence: sndhakal@vcu.edu

Received: 8 January 2020; Accepted: 5 February 2020; Published: 13 February 2020

Table S1. Sequences of oligonucleotides used in constructing lysozyme-specific aptasensor. All biotin- and fluorophore-modified DNA strands were HPLC purified when purchased. Sequences that form the bulge (internal loop of the aptasensor in closed conformation) are bolded. 30 nucleotides aptamer sequence[1] is underlined. 'TEG' represents tetraethyleneglycol spacer between biotin and DNA, which was incorporated to facilitate the unrestricted binding of aptasensors to the streptavidin on the microscope slide.

Strand Name	Sequence (5'-3')
Cy3 strand	Biotin-TEG/TGG AAC TCA CTA CTC **GAT TAG TGT ATG ACC** TCT ATA TGA GAG CTT CTG AT/Cy3
Cy5 strand	Cy5/TAT AGA **ATT ATA TTA TAT TA**C GAG TAG TGA GTT CCA
Top strand	ATC AGA AGC TCT C
B1	AGC CCT GAT GAC AGT AAT ATA ATT CTA TA
H1	TAT AGA ATT ATA TTA CTG TCA TCA GGG CT
Lysozyme Aptamer	ACT GTC <u>ATC AGG GCT AAA GAG TGC AGA GTT ACT TAG</u>

Table S2. Thermal annealing program used to assemble aptasensor. Thermal annealing was carried out by ramping the temperature of the solution from 95 °C to 4 °C in a thermal cycler as described [2,3].

Temperature (°C)	Time (min)
95	5
93	5
90	5
88	5
86	5
84	5
82	5
80	5
78	5
76	5
72	5
68	5
64	5
60	5
56	5
52	5
48	5
44	5
40	5
36	5

32	5
28	5
24	5
4	hold

Table 3. Comparison of analytical performance of our aptasensor with other published methods. Detection amount of lysozyme was calculated based on the detection limit and sample volume per analysis. The detection amount of this work is similar to other fluorescence-based methods. LOD = Limit of Detection. ND = Not Determined (volume per analysis was not available).

Method	Sensing System	Linear Range	LOD	Detection Amount	Reference
Single-Molecule FRET	DNA aptamer	10 nM - 2μM	30 nM	2.3 picomole	This work
Fluorescence	CdTe QDs/aptamer	8.9 nM - 71.2 nM	4.3 nM	4.3 picomole	[4]
Fluorescence	CuInS2 QDs/PDAD cationic polyelectrolyte/aptamer	40 nM - 100 nM	20 nM	14 picomole	[5]
Fluorescence	DNA polymerase/aptamer	0.8 nM – 20 nM	0.8 nM	32 femtomole	[6]
Phosphorescence	Mn-ZnS QDs/β-cyclodextrin/ aptamer	5.5 nM - 44.5 nM	0.5 nM	2.7 picomole	[7]
Electrochemical	MWCNT/Chit/Graphene oxide- AuNP nanocomposite/ aptamer	20 fM - 250 pM	9 fM	ND	[8]
Electrochemical	Amino-rGO/IL/Amino-MSNs nanocomposite/aptamer	10 fM - 200 nM	2.1 fM	63 zeptomole	[9]
Electrochemical	aptamer	1.67 μM - 5 μM	1.7 μM	ND	[10]
SERS	NESA/SDA/HA-EXPAR multiple amplifications/aptamer	1 pM – 1 fM	1 fM	1 zeptomole	[11]

Abbreviations:

FRET: Fluorescence Resonance Energy Transfer
CdTe QDs: cysteamine capped CdTe quantum dots
CuInS2 QDs: Cu-In-S ternary Quantum Dots
PDAD: Poly(DimethylDiallyl) Ammonium chloride
Mn-ZnS QDs: Mn-doped ZnS Quantum Dots
MWCNT: Multi-Walled Carbon NanoTubes
AuNP: Gold NanoParticles
Amino-rGO: Amino-reduced Graphene Oxide
IL: Ionic Liquid
Amino-MSNs: Amino-MesoSilica Nanoparticles
SERS: Surface-enhanced Raman Scattering
NESA: Nicking Enzyme Signaling Amplification
SDA: Strand Displacement Amplification
HA-EXPAR: circular-Hairpin-Assisted EXPonential Amplification Reaction

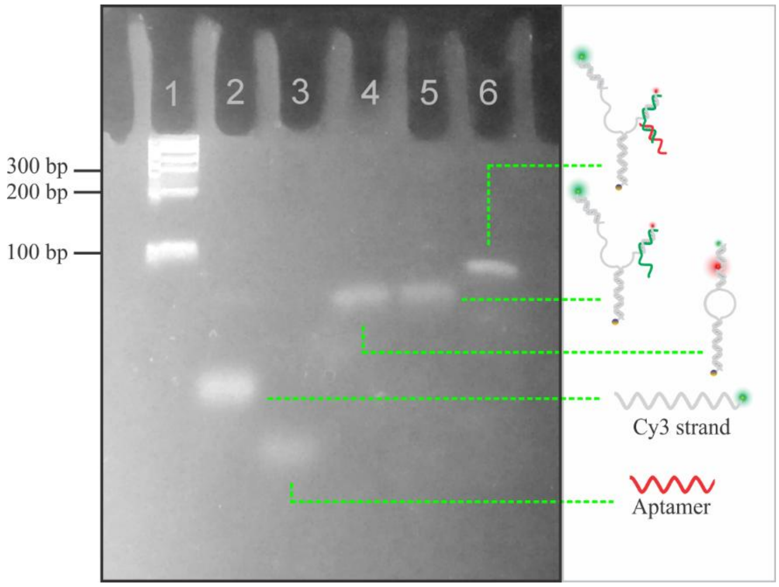

Figure S1. Native PAGE gel characterization of aptasensor assembly. The formation of lysozyme aptasensor was confirmed by running a 7.5% polyacrylamide gel electrophoresis for 1 hr 45 min at 50 V. The gel was stained in an ethidium bromide solution for 20 min before taking an image under UV-Vis transilluminator. The corresponding strand or nanoassembly for each band on lane 1 to 6 is shown. *Lane 1*: molecular weight marker; *Lane 2*: Cy3 strand (50 nt); *Lane 3*: lysozyme aptamer (36 nt); *Lane 4*: partial assembly in the absence of B1 strand and aptamer; *Lane 5*: partial assembly in the absence of aptamer; *Lane 6*: fully assemble aptasensor. The slowest migration (highest molecular weight, 136 nt altogether) of aptasensor compared to all other controls (*Lane 1-5*) confirms the successful assembly of aptasensor.

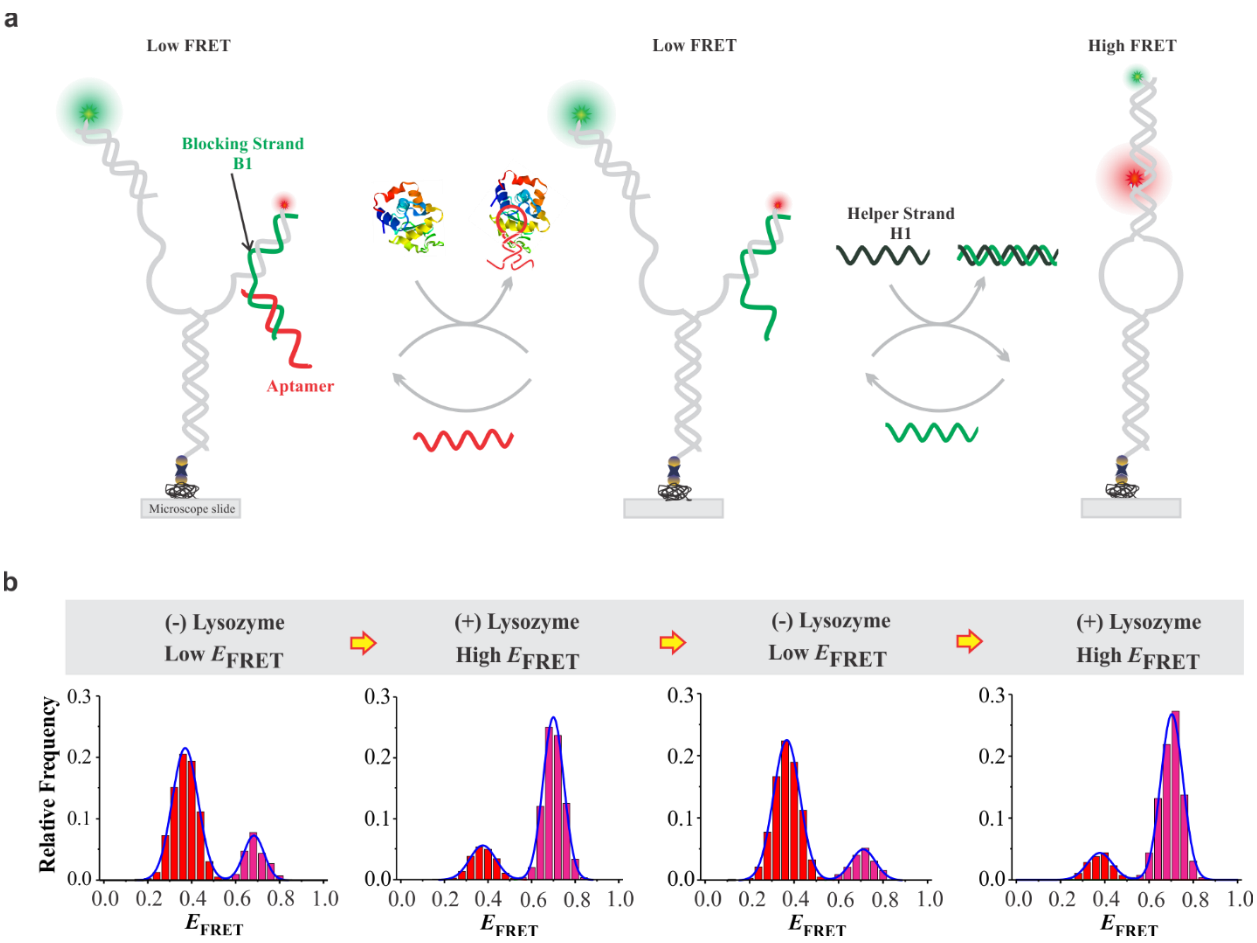

Figure S2. Recyclability of lysozyme aptasensor. (a) Working principle of lysozyme aptasensor illustrating its recyclability. The toehold region on strand B1 becomes available as soon as the lysozyme displaces the aptamer. Consequently, the strand H1 displaces B1 from the aptasensor and therefore results into a closed conformation of aptasensor. (**b**) smFRET analysis of the sensor recyclability. The E_{FRET} histograms show an efficient switching between the low- and high-E_{FRET} states as designed in panel (a). Experiments were performed at the saturating concentration of lysozyme (2 µM) and H1 (1 µM) whereas recycling was done using 1 µM solution of each H1 strand and aptamer to ensure an efficient switching.

References

1. Cox, J. C.; Ellington, A. D. Automated Selection of Anti-Protein Aptamers. *Bioorg. Med. Chem.* 2001, 9 (10), 2525–2531.
2. Kaur, A.; Sapkota, K.; Dhakal, S. Multiplexed Nucleic Acid Sensing with Single-Molecule FRET. *ACS Sens.* **2019**, *4*(3), 623–633.
3. Megalathan, A.; Cox, B.D.; Wilkerson, P.D.; Kaur, A.; Sapkota, K.; Reiner, J.E.; Dhakal, S. Single-Molecule Analysis of i-Motif within Self-Assembled DNA Duplexes and Nanocircles. Nucleic Acids Res. **2019**, 47 (14), 7199–7212.
4. Li, S.; Gao, Z.; Shao, N. Non-Covalent Conjugation of CdTe QDs with Lysozyme Binding DNA for Fluorescent Sensing of Lysozyme in Complex Biological Sample. *Talanta* **2014**, *129*, 86–92.
5. Liu, S.; Na, W.; Pang, S.; Shi, F.; Su, X. A Label-Free Fluorescence Detection Strategy for Lysozyme Assay Using CuInS2 Quantum Dots. *Analyst* **2014**, *139* (12), 3048–3054.
6. Jung, Y.; Lee, C. Y.; Park, K. S.; Park, H. G. Sensitive and Specific Detection of Proteins Based on Target-Responsive DNA Polymerase Activity. *Anal. Chim. Acta* **2019**, *1059*, 80–85.
7. Zuo, L.; Qin, G.; Lan, Y.; Wei, Y.; Dong, C. A Turn-on Phosphorescence Aptasensor for Ultrasensitive Detection of Lysozyme in Humoral Samples. *Sens. Actuators B Chem.* **2019**, *289*, 100–105.
8. Heydari-Bafrooei, E.; Askari, S. Ultrasensitive Aptasensing of Lysozyme by Exploiting the Synergistic Effect of Gold Nanoparticle-Modified Reduced Graphene Oxide and MWCNTs in a Chitosan Matrix. *Microchim. Acta* **2017**, *184* (9), 3405–3413.
9. Jamei, H. R.; Rezaei, B.; Ensafi, A. A. An Ultrasensitive Electrochemical Anti-Lysozyme Aptasensor with Biorecognition Surface Based on Aptamer/Amino-RGO/Ionic Liquid/Amino-Mesosilica Nanoparticles. *Colloids Surf. B Biointerfaces* **2019**, *181*, 16–24.
10. Ortiz-Aguayo, D.; Del Valle, M. Label-Free Aptasensor for Lysozyme Detection Using Electrochemical Impedance Spectroscopy. *Sensors* **2018**, *18* (2), 354.
11. He, P.; Zhang, Y.; Liu, L.; Qiao, W.; Zhang, S. Ultrasensitive SERS Detection of Lysozyme by a Target-Triggering Multiple Cycle Amplification Strategy Based on a Gold Substrate. *Chem. – Eur. J.* **2013**, *19* (23), 7452–7460.
